# Author Correction: TERRA transcription destabilizes telomere integrity to initiate break-induced replication in human ALT cells

**DOI:** 10.1038/s41467-023-39584-1

**Published:** 2023-06-28

**Authors:** Bruno Silva, Rajika Arora, Silvia Bione, Claus M. Azzalin

**Affiliations:** 1 Instituto de Medicina Molecular João Lobo Antunes (iMM), Faculdade de Medicina da Universidade de Lisboa, Lisbon, Portugal; 2 Computational Biology Unit, Institute of Molecular Genetics Luigi Luca Cavalli-Sforza, National Research Council, Pavia, Italy; 3 Present Address: Institute for Molecular Health Sciences, ETHZ, Zürich, Switzerland

**Keywords:** Telomeres, Long non-coding RNAs

Correction to: *Nature Communications* 10.1038/s41467-021-24097-6, published online 18 June 2021

In the Acknowledgements section of this article, one funding source was incorrectly cited as PTDC/BIA-MOL/29352/2017; the correct citation for the funding should have been ‘LISBOA-01-0145-FEDER-0 29352, project co-funded by FEDER, through POR Lisboa 2020 - Programa Operacional Regional de Lisboa and Fundação para a Ciência e a Tecnologia’. The original article has been corrected.

