# Supplementary material for: TERRA transcription destabilizes telomere integrity to initiate break-induced replication in human ALT cells
Source: Nat Commun. 2021 Jun 18;12:3760. doi: 10.1038/s41467-021-24097-6 (PMC8213692; doi:10.1038/s41467-021-24097-6)
Supplement: Supplementary file 1 — Supplementary Information [file 41467_2021_24097_MOESM1_ESM.pdf]

Supplementary information for:

TERRA transcription destabilizes telomere integrity to initiate break-induced replication in human ALT cells.

Bruno Silva, Rajika Arora, Silvia Bione and Claus M. Azzalin

SUPPLEMENTARY FIGURES

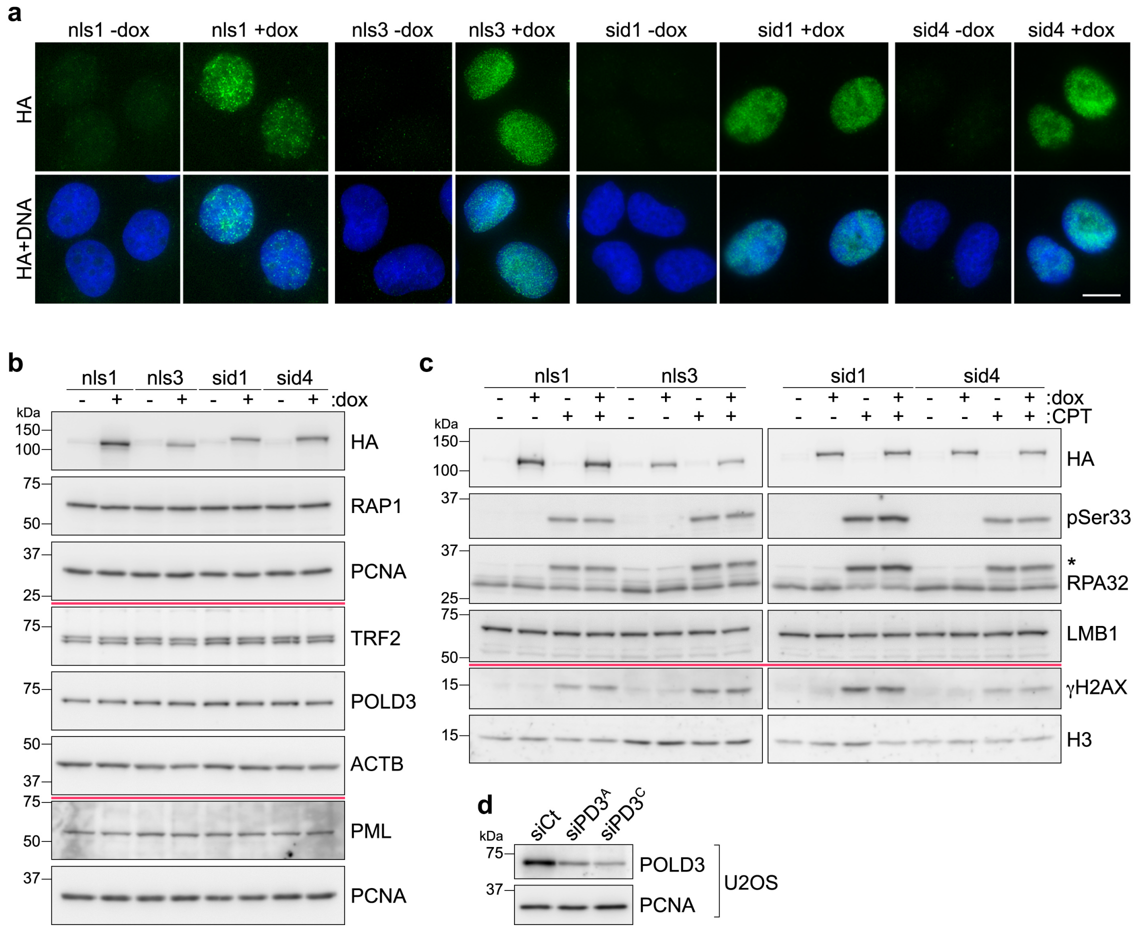

Supplementary Figure 1: Expression of T-TALEs and endogenous proteins used in this study. (**a**) Examples of anti-HA tag IF (green) in the indicated cell lines treated with dox for 24 hours or left untreated. DAPI stained DNA is in blue. Scale bar: 10 µm. (**b**) Western blot analysis of proteins from the indicated cell lines treated with dox as in **a**. Beta actin (ACTB) and PCNA serve as loading controls. (**c**) Western blot analysis of proteins from the indicated cell lines treated with dox as in **a** and with camptothecin (CPT) for 6 hours. Lamin B1 (LMB1) and histone H3 serve as loading controls. The red lines separate images from different membranes. (**d**) Western blot analysis of T-Rex-U2OS cells transfected with two independent siRNAs against POLD3 (siPD3a and siPD3c) or with control siRNAs (siCt). Cells were harvested 6 days after transfection. PCNA serves as loading control. Images are representative of experiments repeated at least twice. Source data are provided as a Source Data file.

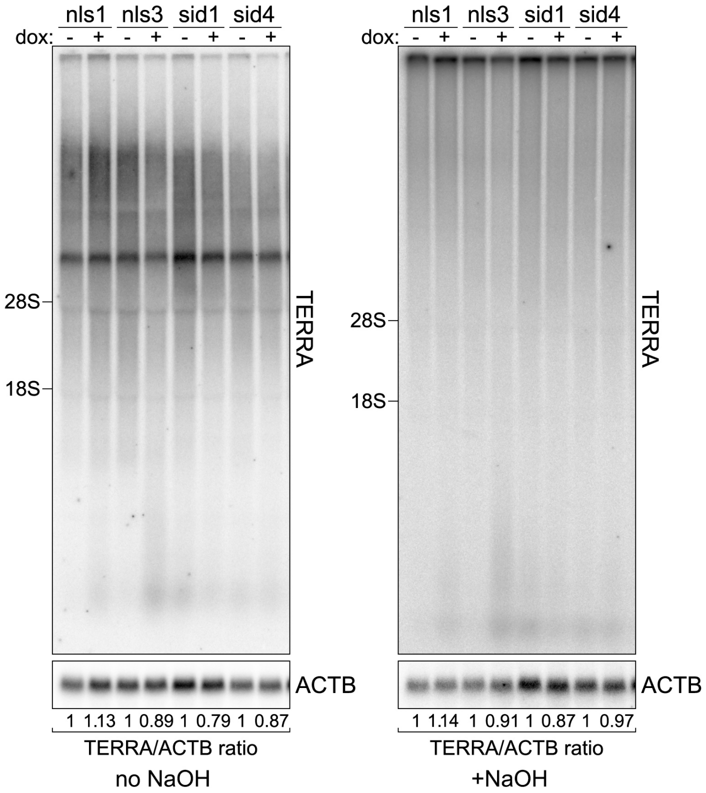

Supplementary Figure 2: Effects of T-TALE expression on cellular UUAGGG pools. TERRA northern blots using total RNA from the indicated cells lines treated with dox for 24 hours or left untreated. For the gel on the right, RNA was treated with NaOH prior to blotting to favor transferring of long TERRA molecules. The positions of 28S and 18S rRNAs are indicated on the left of each blot. Beta Actin (ACTB) mRNA serves as loading control. Numbers at the bottom are the ratios between the total TERRA signal and the one of ACTB and relative to untreated cells. Images are representative of experiments repeated three times. Source data are provided as a Source Data file.

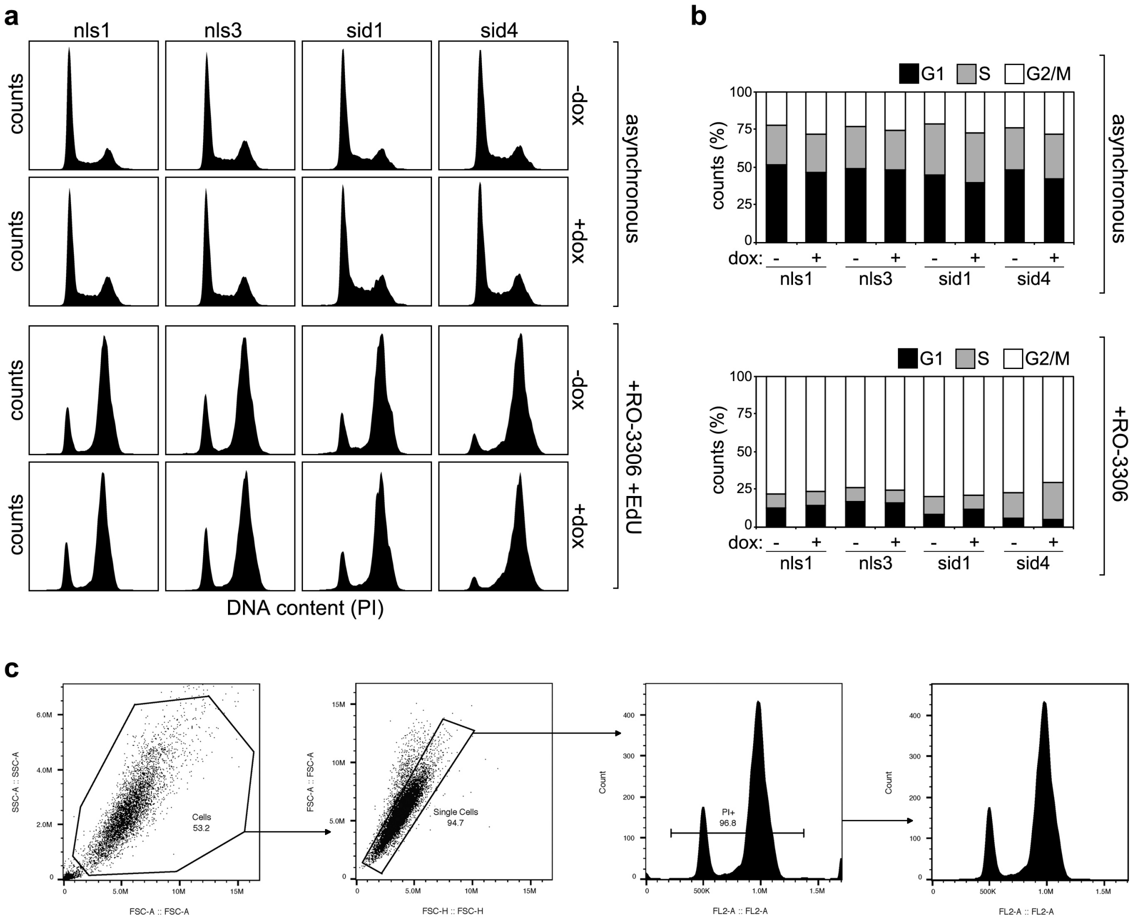

Supplementary Figure 3: Cell cycle distribution analysis of T-TALE cells. (**a**) FACS profiles of the indicated propidium iodide (PI)-stained cells treated or not with dox. Asynchronous cells (upper panels) were used for detection of RPA32, pSer33, gH2AX (Fig. 2) and APBs (Fig. 3); cells treated with the CDK1 inhibitor RO-3306 and EdU (lower panels) were used for detection of EdU incorporation at telomeres and POLD3 (Fig. 3). For Cell counts (y axis) are plotted against PI intensity (x axis). Cells were harvested after 24 (asynchronous) or 24.5 (RO-3306-treated) hours of dox treatment (see methods for details). (**b**) Quantifications of experiments as in **a**. The graphs show the percentage of cells in G1, S and G2/M phases from one representative experiment. (**c**) Gating strategy applied for the analysis. The example corresponds to the bottom left sample in **a**.

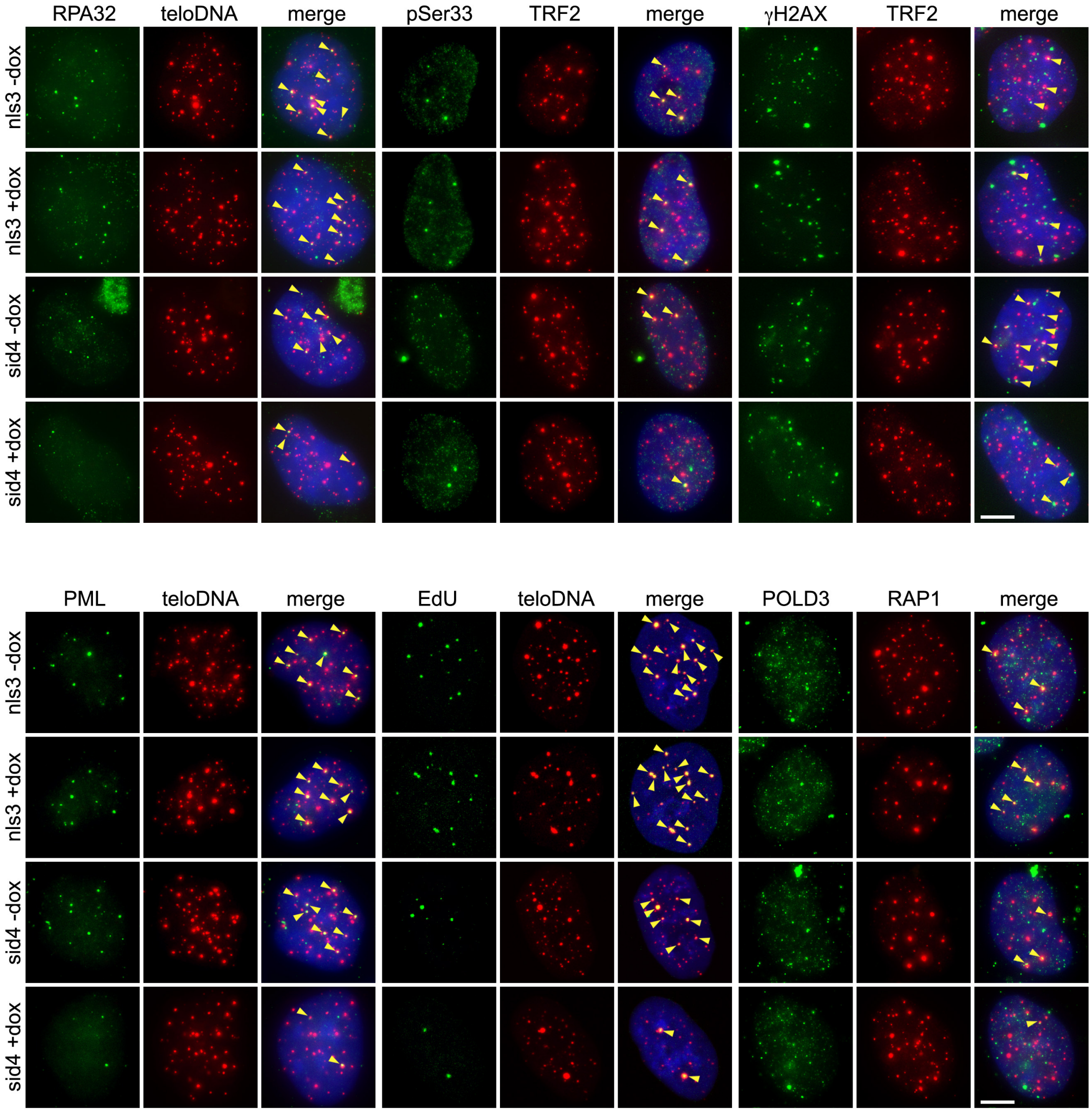

Supplementary Figure 4: TERRA transcription inhibition alleviates telomere instability and ALT activity. Examples of experiments as in Figures 2 and 3 performed in nls3 and sid4 cells. Markers and DAPI stained DNA are shown with the same colors as in Figures 2 and 3. Arrowheads in the merge panels point to co-localization events. Scale bars: 5 μm.

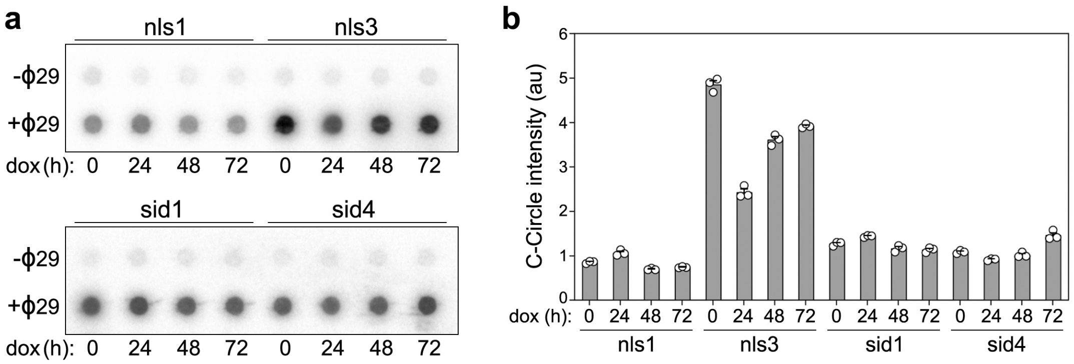

Supplementary Figure 5: C-circle analysis in T-TALE cells. (**a**) C-circle assay analysis of genomic DNA from the indicated cells treated with dox for up to 72 hours. Reaction products were dot-blotted and hybridized with a radiolabeled telomeric probe. Control reactions were performed in absence of phi29 polymerase (-Φ29). (**b**) Quantifications of C-circle signals from experiments as in **a**. Bars and error bars are means and SEMs from 3 independent experiments. Circles are single data points. Source data are provided as a Source Data file.

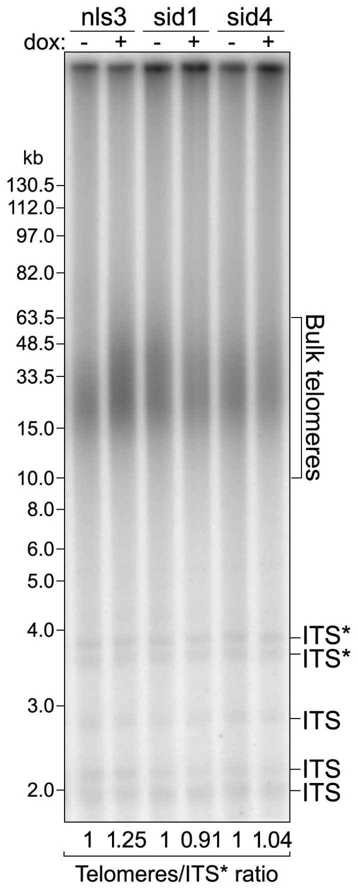

Supplementary Figure 6: Effects of T-TALE expression on bulk telomeres. PFGE TRF analysis of genomic DNA from the indicated cell lines treated with dox for 15 days or left untreated. ITS: intrachromosomal telomeric sequences. Numbers at the bottom are the ratios between the bulk telomere signal and the one of the ITSs indicated by asterisks and relative to untreated cells. Numbers on the left are molecular weights in kb. The image is representative of experiments repeated three times. Source data are provided as a Source Data file.

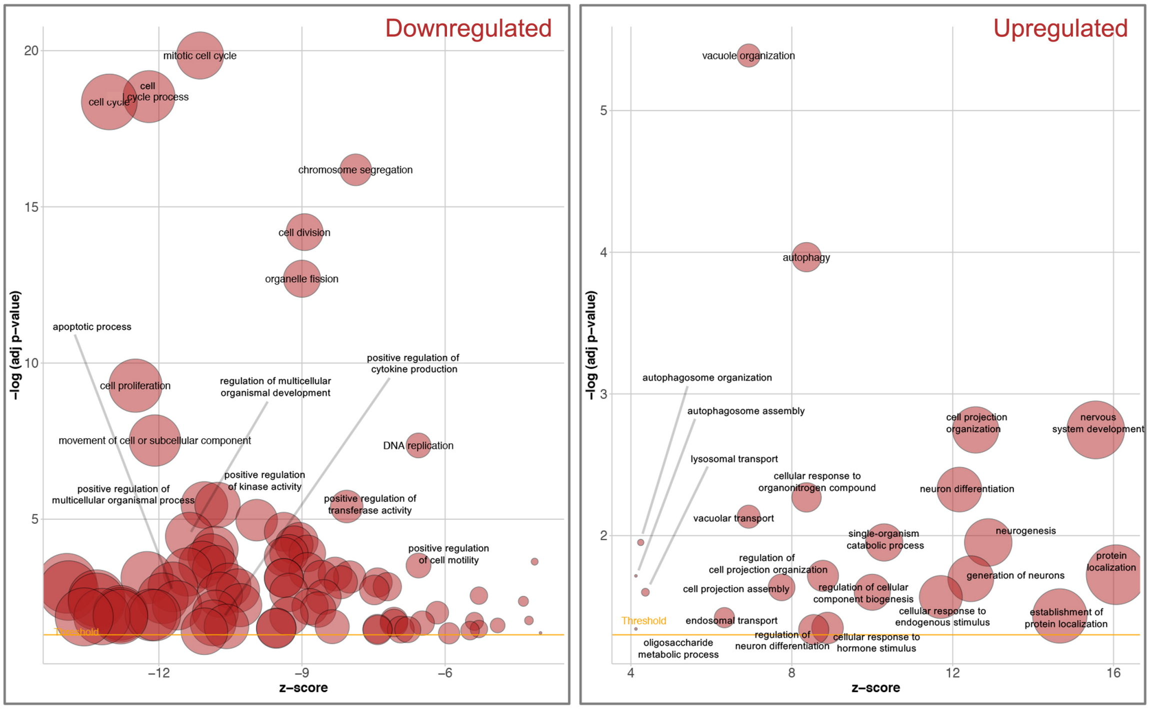

Supplementary Figure 7: Bubble plot representation of the Gene Ontology (GO) analysis of down- (left panel) and up-regulated (right panel) genes in sid4 cells treated with dox for 72 hours versus untreated cells. Significantly enriched biological process (BP) functional categories (FDR *P* ≤ 0.05) are represented as bubbles and the statistical significance of each term is indicated on the Y-axis by the -log10 of the Benjanimi-Hochberg corrected *P*-value (adj p-value). On the X-axis, the z-score of expression value is represented. Bubble areas directly correlate with the number of differentially expressed genes annotated in each GO-BP term. The names of selected GO-BP terms are indicated. The full list of GO-BP terms can be found in Table S3.

Supplementary Table 1: oligonucleotides used in this study.

Name	Oligo sequence (5'-3')	Genomic locus	Application	Amplicon position from the TALE target (bp)	Amplicon position to the first telomeric repeat (bp)
9pXYqnearF	TGTCCTCTGCACAGATTTCG	9pXqYq subtelomere	ChIP qPCR, TERRA RT-qPCR	84-248 (9p)<br>76-240 (XqYq)	396-560 (9p)<br>284-448 (Xq)<br>284-448 (Yq)
9pXYqnearR	TCTGTGCTTAGGGGAATGCT				
9pXYqfarF	TTCCGCACTGAACCGCTCTAA	9pXqYq subtelomere	ChIP qPCR, TERRA RT-qPCR	318-369 (9p)<br>311-362 (Xq)	275-326 (9p)<br>162-213 (Xq)<br>162-213 (Yp)
9pXYqfarR	GCAGCCATGAATAATCAAGGT				
10qnearF	TAGCACACACCCGGAGAGCA	10q subtelomere	ChIP qPCR	34-118	690-774
10qnearR	CTCTGCTCCGCCTTCGCAAT				
10qfarF	GAATCCTGCGCACCGAGAT	10q subtelomere	ChIP qPCR, TERRA RT-qPCR	384-448	360-424
10qfarR	CTGCACTTGAACCCTGCAATAC				
15q16pnearF	GCCTTTGCGACGGCGGAG	15q subtelomere	ChIP qPCR	6-112 (15q)<br>8-113 (16p)	459-565 (15q)<br>434-539 (16p)
15q16pnearR	CGCCTTCGCAGTACCACC				
15qfarF	CAGCGAGATTCTCCCAAGCTAAG	15q subtelomere	ChIP qPCR, TERRA RT-qPCR	395-530	41-176
15qfarR	AACCCTAACCACATGAGCAACG				
16pfarF	TGTGTTTCAACGCTGCAACTG	16p subtelomere	ChIP qPCR, TERRA RT-qPCR	341-463	84-206
16pfarR	AGTTAGAACGGTTCAGTGTG				
10p18pF	CCTTCTAACTGGACTCTGAC	10p subtelomere	ChIP qPCR, TERRA RT-qPCR	na	8-80 (10p)<br>8-80 (18p)
10p18pR	GCCACAGCGACGGTAAATAA				
12qF	ATTTCCCGTTTTCCACACTGA	12q subtelomere	ChIP qPCR, TERRA RT-qPCR	na	346-413
12qR	CTGTTTGCAGCGCTGAATATTC				
20qF	GCAGCTTTCTCAGCACAC	20q subtelomere	ChIP qPCR, TERRA RT-qPCR	na	34-109
20qR	TTTGTTCACTGTCGATGCG				
XYpF	GCAAAGAGTGAAAGAACGAAGCTT	XpYp subtelomere	ChIP qPCR, TERRA RT-qPCR	na	1-136
XYpR	CCCTCTGAAAGTGGACCAATCA				
XcenFwd	GTGACGATGGAGTTTAACTCAGGG	X centromere	ChIP qPCR	na	na
XcenRev	GCTTTCCGTTCAGTTATGGGAAGG				
AlphoidF	CTCAGAAACTTCTTTGTGATGTGT	Aplhoid DNA	ChIP qPCR	na	na
AlphoidR	TATTCCCTTTTGGAACGAAGGC				

ActF	TCCCTGGAGAAGAGCTACGA	Beta Actin gene	ChIP qPCR, TERRA RT-qPCR	na	na
ActR	AGCACTGTGTTGGCGTACAG				
U6F	CTCGCTTCGGCAGCACATATA	U6 gene	ChIP qPCR	na	na
U6R	GGAACGCTTCACGAATTTGCGT				
TeloR	(CCCTAA)5	Telomeric repeats	TERRA RT-qPCR	na	na
Actin_2	GTGAGGATCTTCATGAGGTAGTCAGTCAGGT	Beta Actin gene	Northern Blot	na	na

na: not applicable.
