# Supplementary material for: TERRA transcription destabilizes telomere integrity to initiate break-induced replication in human ALT cells
Source: Nat Commun. 2021 Jun 18;12:3760. doi: 10.1038/s41467-021-24097-6 (PMC8213692; doi:10.1038/s41467-021-24097-6)
Supplement: Supplementary file 3 — Description of Additional Supplementary Files [file 41467_2021_24097_MOESM3_ESM.pdf]

Description of Additional Supplementary Files

File Name: Supplementary Data 1
Description: (**a**) Differential Expression Analysis results of the comparison of dox treated and untreated sid4 cells. (**b**) Differential Expression Analysis results of the comparison of dox treated and untreated nls3 cells. (**c**) Summary of the results of Differential Expression Analyses.

File Name: Supplementary Data 2
Description: (**a**) Gene Ontology Biological Process functional enrichment analysis of the genes differentially expressed in the comparison between dox-treated (72 hours) and untreated sid4 cells. (**b**) Significant Gene Ontology terms grouped on the basis of their functional relationship. (**c**) Gene Ontology Biological Process functional enrichment analysis of the genes up-regulated in the comparison between dox treated and untreated sid4 cells. (**d**) Gene Ontology Biological Process functional enrichment analysis of the genes down-regulated in the comparison between dox treated and untreated sid4 cells.
