# Supplementary material for: TERRA transcription destabilizes telomere integrity to initiate break-induced replication in human ALT cells
Source: Nat Commun. 2021 Jun 18;12:3760. doi: 10.1038/s41467-021-24097-6 (PMC8213692; doi:10.1038/s41467-021-24097-6)
Supplement: Supplementary file 6 — Reporting Summary [file 41467_2021_24097_MOESM6_ESM.pdf]

nature research

Corresponding author(s): Claus M. Azzalin

Last updated by author(s): May 6, 2021

Reporting Summary

Nature Research wishes to improve the reproducibility of the work that we publish. This form provides structure for consistency and transparency in reporting. For further information on Nature Research policies, see our Editorial Policies and the Editorial Policy Checklist.

Statistics

For all statistical analyses, confirm that the following items are present in the figure legend, table legend, main text, or Methods section.

n/a	Confirmed	
☐	☒	The exact sample size (*n*) for each experimental group/condition, given as a discrete number and unit of measurement
☐	☒	A statement on whether measurements were taken from distinct samples or whether the same sample was measured repeatedly
☐	☒	The statistical test(s) used AND whether they are one- or two-sided<br>*Only common tests should be described solely by name; describe more complex techniques in the Methods section.*
☒	☐	A description of all covariates tested
☒	☐	A description of any assumptions or corrections, such as tests of normality and adjustment for multiple comparisons
☐	☒	A full description of the statistical parameters including central tendency (e.g. means) or other basic estimates (e.g. regression coefficient) AND variation (e.g. standard deviation) or associated estimates of uncertainty (e.g. confidence intervals)
☐	☒	For null hypothesis testing, the test statistic (e.g. *F*, *t*, *r*) with confidence intervals, effect sizes, degrees of freedom and *P* value noted<br>*Give P values as exact values whenever suitable.*
☒	☐	For Bayesian analysis, information on the choice of priors and Markov chain Monte Carlo settings
☒	☐	For hierarchical and complex designs, identification of the appropriate level for tests and full reporting of outcomes
☒	☐	Estimates of effect sizes (e.g. Cohen's *d*, Pearson's *r*), indicating how they were calculated

Our web collection on statistics for biologists contains articles on many of the points above.

Software and code

Policy information about availability of computer code

Data collection	Rotor-Gene 6000 Series Software (v.1.7), Amersham Typhoon Scanner (v.2.0.0.6), Amersham Imager 680 (v.2.0.0), ZEN (v.3.0).
Data analysis	ImageJ (v1.52p), Adobe photoshop (v.13.0), Microsoft Excel (Office 2016), Graph Pad Prism (v.8.4.3), FlowJo (v.10.7.2), FastQC (v.0.11.8), RNA STAR (v.2.7.7a), featureCounts (v.2.0.1), DESeq2 (v.2.11.40.6), DAVID (v.6.8), R packages ggplot2 (v3.3.2) and GOplot (v1.0.2).

For manuscripts utilizing custom algorithms or software that are central to the research but not yet described in published literature, software must be made available to editors and reviewers. We strongly encourage code deposition in a community repository (e.g. GitHub). See the Nature Research guidelines for submitting code & software for further information.

Data

Policy information about availability of data

All manuscripts must include a data availability statement. This statement should provide the following information, where applicable:

- Accession codes, unique identifiers, or web links for publicly available datasets
- A list of figures that have associated raw data
- A description of any restrictions on data availability

All raw RNA sequencing data are publicly available through the NCBI Sequence Read Archive (BioProject ID: PRJNA699729; https://submit.ncbi.nlm.nih.gov/subs/sra/SUB9021336/overview).

Field-specific reporting

Please select the one below that is the best fit for your research. If you are not sure, read the appropriate sections before making your selection.

☒ Life sciences ☐ Behavioural & social sciences ☐ Ecological, evolutionary & environmental sciences

For a reference copy of the document with all sections, see nature.com/documents/nr-reporting-summary-flat.pdf

Life sciences study design

All studies must disclose on these points even when the disclosure is negative.

Sample size	No statistical method was used to pre-determine sizes. The numbers of independent experiments (n) are indicated in the Methods section and in figure legends. Samples sizes and statistical tests were selected based on previous studies with similar methodologies (PMIDs: 31138795 and 29358759).
Data exclusions	No data were excluded from the analysis.
Replication	All experiments were reliably reproduced. At least three independent biological replicates were performed unless clearly stated in the figure legend.
Randomization	No randomization was used based on previous studies with similar methodologies (PMIDs: 31138795 and 29358759).
Blinding	For collection of microscopy images nuclei were selected only based on DAPI staining; no blinding was used for data analysis based on previous studies with similar methodologies (PMIDs: 31138795 and 29358759).

Reporting for specific materials, systems and methods

We require information from authors about some types of materials, experimental systems and methods used in many studies. Here, indicate whether each material, system or method listed is relevant to your study. If you are not sure if a list item applies to your research, read the appropriate section before selecting a response.

Materials & experimental systems

n/a	Involved in the study	
☐	☒	Antibodies
☐	☒	Eukaryotic cell lines
☒	☐	Palaeontology and archaeology
☒	☐	Animals and other organisms
☒	☐	Human research participants
☒	☐	Clinical data
☒	☐	Dual use research of concern

Methods

n/a	Involved in the study	
☒	☐	ChIP-seq
☐	☒	Flow cytometry
☒	☐	MRI-based neuroimaging

Antibodies

Antibodies used	Primary antibodies: a rabbit monoclonal anti-HA (Cell Signaling, 3724), a rabbit polyclonal anti-RAP1 (Bethyl, A300-306A), a mouse monoclonal anti-PCNA (Santa Cruz Biotechnology, sc-56), a rabbit polyclonal anti-TRF2 (Novus Biologicals, NB110-57130), a mouse monoclonal anti-TRF2 (Millipore, 05-521), a mouse monoclonal anti-POLD3 (Novus Biologicals, H00010714-M01), a mouse monoclonal anti-ACTB (Santa Cruz Biotechnology, sc-47778), a mouse monoclonal anti-PML (Santa Cruz Biotechnology, sc-966), a rabbit polyclonal anti-pSer33 (Bethyl, A300-246A), a rabbit polyclonal anti-RPA32 (Bethyl, A300-244A), a rabbit polyclonal anti-LMB1 (GeneTex, GTX103292S), a rabbit polyclonal anti-H3 (Santa Cruz Biotechnology, sc-10809), a mouse monoclonal anti-gH2AX (Millipore, 05-636), a rabbit polyclonal anti-BLM (Bethyl Laboratories, A300-110A), a rabbit polyclonal anti-HROB (Atlas Antibodies, HPA023393), a mouse monoclonal anti-BRCA1 (Santa Cruz Biotechnology, sc-6954), a mouse monoclonal anti-EXO1 (Santa Cruz Biotechnology, sc-56092), a mouse monoclonal anti-FANCD2 (Santa Cruz Biotechnology, sc-20022), a rabbit polyclonal anti-GEN1 (Atlas Antibodies, HPA020078), a rabbit polyclonal antiRAD51AP1 (GeneTex, GTX115455), a mouse monoclonal anti-BRCA2 (Sigma-Aldrich, OP95), a mouse monoclonal anti-ORC1 (Santa Cruz Biotechnology, sc-398734), a rat monoclonal anti-ORC6 (Santa Cruz Biotechnology, sc-32735), a rabbit monoclonal anti-LIG4 (Abcam, ab193353). Secondary antibodies: HRP-conjugated goat anti-mouse (Bethyl Laboratories, A90-116P), anti-rabbit (Bethyl Laboratories, A120-101P) and anti-rat (Santa Cruz Biotechnology, sc-2006) IgGs; Alexa Fluor 568-conjugated donkey anti-rabbit IgGs (Thermo Fisher Scientific, A10042) and Alexa Fluor 488-conjugated donkey anti-mouse IgGs (Thermo Fisher Scientific, A21202).
Validation	Rabbit monoclonal anti-HA (Cell Signaling, 3724) was validated in this work in samples overexpressing the transgenes upon doxycycline induction; mouse monoclonal anti-POLD3 (Novus Biologicals, H00010714-M01) was validated in this work on siRNA treated samples; rabbit polyclonal anti-pSer33 (Bethyl, A300-246A), rabbit polyclonal anti-RPA32 (Bethyl, A300-244A) and mouse monoclonal anti-gH2AX (Millipore, 05-636) were validated in this work by using samples where DNA damage was induced by treatment with camptothecin; rabbit polyclonal anti-BLM (Bethyl Laboratories, A300-110A) was validated in Silva et al. 2019 (PMID:

31138795) on siRNA treated samples; mouse monoclonal anti-PML (Santa Cruz Biotechnology, sc-966) was validated in Loe et al. 2020 (PMID: 32217664) on knockout samples; rabbit polyclonal anti-RAP1 (Bethyl, A300-306A) was validated in Lototska et al. 2020 (PMID: 32096305) on shRNA treated samples; rabbit polyclonal anti-TRF2 (Novus Biologicals, NB110-57130) was validated in Mendez-Bermudez et al. 2018 (PMID: 29727617) on shRNA treated samples; mouse monoclonal anti-TRF2 (Millipore, 05-521) was validated in Lee et al. 2018 (PMID: 29358759) on overexpressing and shRNA treated samples; mouse monoclonal anti-BRCA1 (Santa Cruz Biotechnology, sc-6954) was validated in Wang et al. 2019 on samples naturally lacking BRCA1 protein; mouse monoclonal anti-EXO1 (Santa Cruz Biotechnology, sc-56092) was validated in Thakar et al. 2020 (PMID: 32358495) on siRNA treated samples; mouse monoclonal anti-FANCD2 (Santa Cruz Biotechnology, sc-20022) and mouse monoclonal anti-BRCA2 (Sigma-Aldrich, OP95) were validated in Raghunandan et al. 2015 (PMID: 25659033) on siRNA treated samples; rabbit polyclonal anti-RAD51AP1 (GeneTex, GTX115455) was validated in Park et al. 2019 (PMID: 31844045) on siRNA treated samples; rabbit monoclonal anti-LIG4 (Abcam, ab193353) was validated in Xing et al. 2019 (PMID: 31141305) on siRNA treated samples; rabbit polyclonal anti-HROB (Atlas Antibodies, HPA023393) was validated in Wang et al. 2020 (PMID: 32853826) on shRNA treated samples; mouse monoclonal anti-PCNA (Santa Cruz Biotechnology, sc-56) was validated in Li et al. 2018 (PMID: 30006506) on PCNA overexpressing samples; rabbit polyclonal anti-LMB1 (GeneTex, GTX103292S) was validated by the supplier on knockout samples; mouse monoclonal anti-ACTB (Santa Cruz Biotechnology, sc-47778) - manufacturer´s website validation: Western blot analysis of β-Actin expression in MCF7, NIH/3T3, KNRK, HeLa, Jurkat, Sol8, C32 and 293T whole-cell lysates. Cited in more than 10000 publications. Citations for the antibody are listed at https://www.scbt.com/p/beta-actin-antibody-c4;
rabbit polyclonal anti-H3 (Santa Cruz Biotechnology, sc-10809) - manufacturer´s website validation: Western blot analysis of Histone H3.3B expression in non-transfected and transfected 293T. Cited in 67 publications. Citations for the antibody are listed at https://www.scbt.com/p/histone-h3-antibody-fl-136;
rat monoclonal anti-ORC6 (Santa Cruz Biotechnology, sc-32735) - manufacturer´s website validation: Western blot analysis of ORC6 expression in non-transfected and transfected 293T and in U-2 OS whole cell lysates. Cited in 67 publications. Citations for the antibody are listed at https://www.scbt.com/p/orc6-antibody-3a4
mouse monoclonal anti-ORC1 (Santa Cruz Biotechnology, sc-398734) - manufacturer´s website validation: Western blot analysis of ORC1 expression in HeLa and Ramos nuclear extracts, and in Hep G2 and MCF7 whole cell lysates. Information for the antibody is found at https://www.scbt.com/p/orc1-antibody-f-10.

Eukaryotic cell lines

Policy information about cell lines

Cell line source(s)	T-REx™-U2OS cells were purchased from ThermoFisher Scientific.
Authentication	Authentication was carried out by the ThermoFisher Scientific. ALT features scored in our work further validated the authenticity of the cells.
Mycoplasma contamination	All cells tested negative for mycoplasma contamination using LookOut Mycoplasma PCR Detection Kit (Sigma- Aldrich).
Commonly misidentified lines (See ICLAC register)	U2OS cells are not listed as commonly misidentified at ICLAC.

Flow Cytometry

Plots

Confirm that:

☒ The axis labels state the marker and fluorochrome used (e.g. CD4-FITC).

☒ The axis scales are clearly visible. Include numbers along axes only for bottom left plot of group (a 'group' is an analysis of identical markers).

☒ All plots are contour plots with outliers or pseudocolor plots.

☒ A numerical value for number of cells or percentage (with statistics) is provided.

Methodology

Sample preparation	Cells were trypsinized and pelleted by centrifugation at 500 g at 4°C for 5 min. Cell pellets were fixed in 70% ethanol at -20°C for 30 min and treated with 25 ug/ml RNaseA (Sigma-Aldrich) in 1x PBS at 37°C for 20 min. Cells were then centrifuged as above and pellets washed in 1x PBS and stained with 20 ug/ml propidium iodide (Sigma-Aldrich) in 1x PBS at 4°C for 10 min.
Instrument	BD Accuri C6 (BD Biosciences).
Software	FlowJo (v.10.7.2).
Cell population abundance	No sorting.
Gating strategy	Gating strategy is shown in Supplementary Figure 3c.

☒ Tick this box to confirm that a figure exemplifying the gating strategy is provided in the Supplementary Information.
