# Supplementary material for: TERRA transcription destabilizes telomere integrity to initiate break-induced replication in human ALT cells
Source: Nat Commun. 2021 Jun 18;12:3760. doi: 10.1038/s41467-021-24097-6 (PMC8213692; doi:10.1038/s41467-021-24097-6)

Uncropped blots (related to Fig. 6)

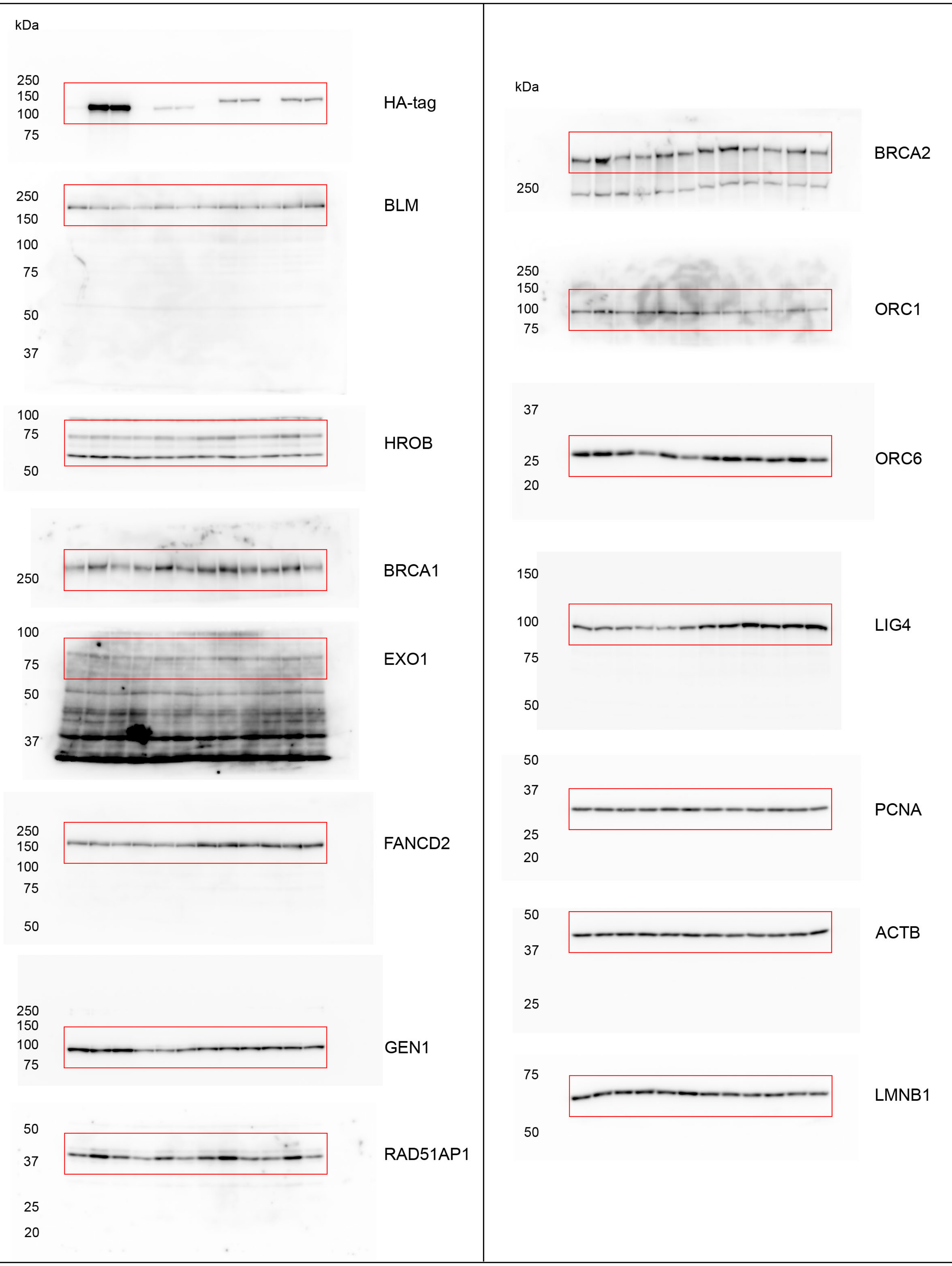

Uncropped blots (related to Supplementary Fig. 1)

Fig. S1b

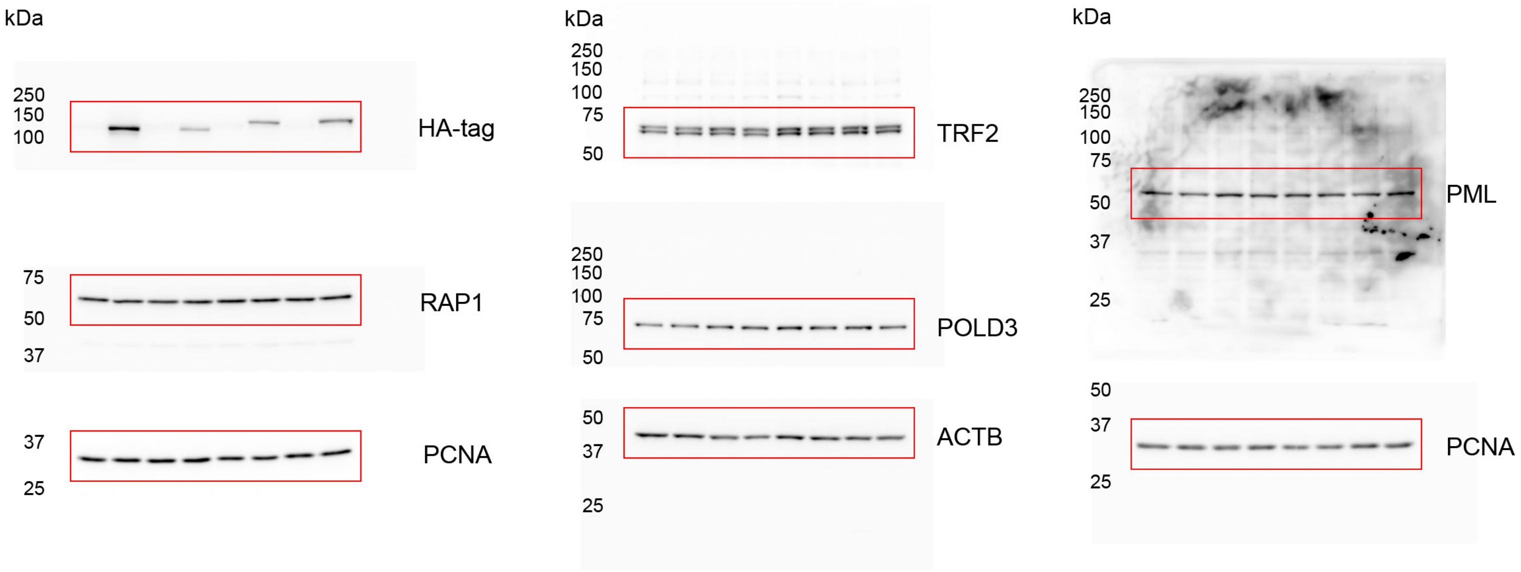

Fig. S1c

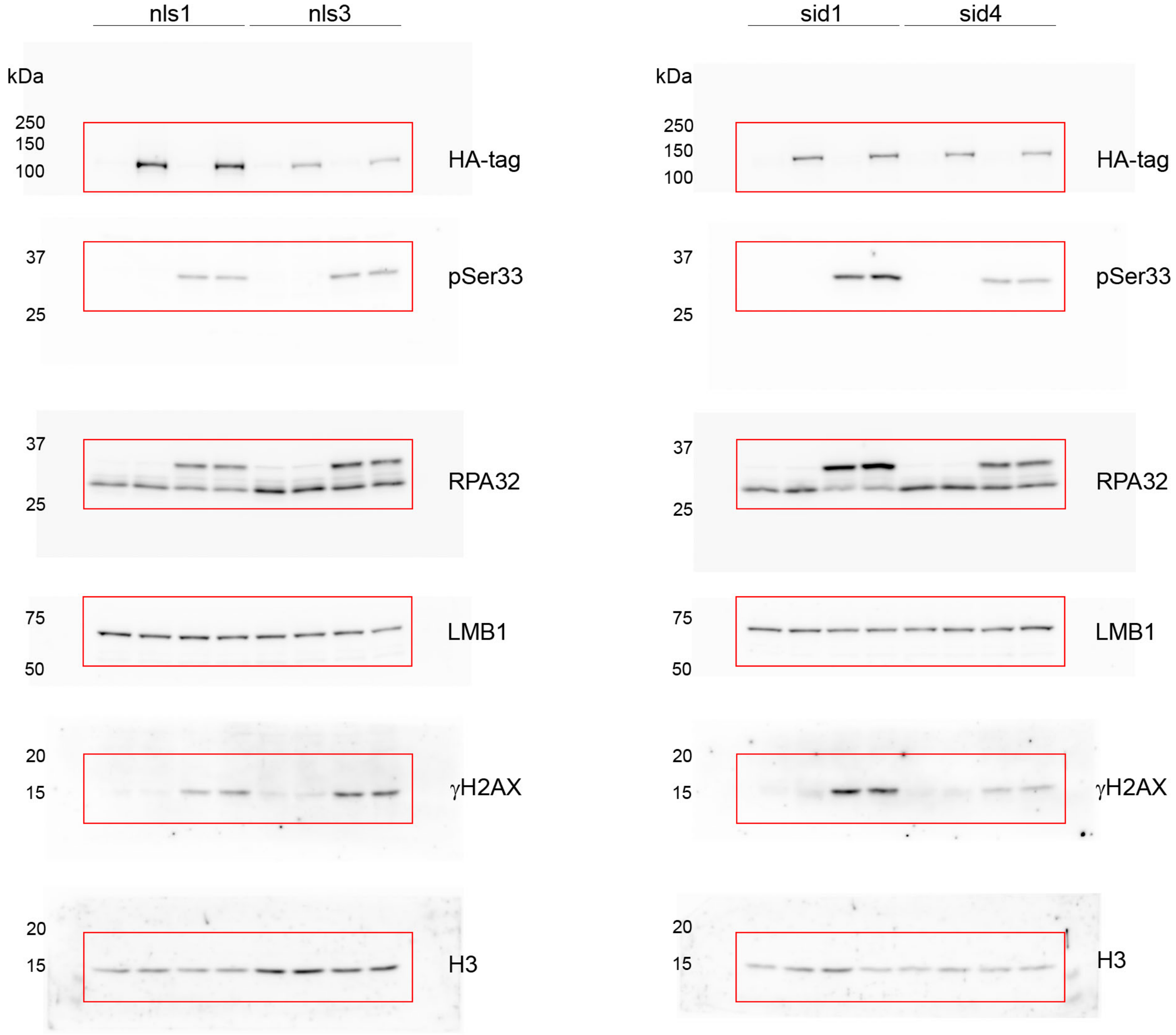

Fig. S1d

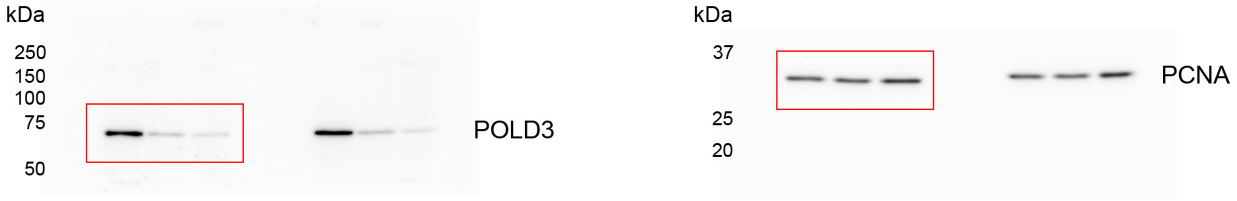

Uncropped blots (related to Supplementary Fig. 2)

no NaOH

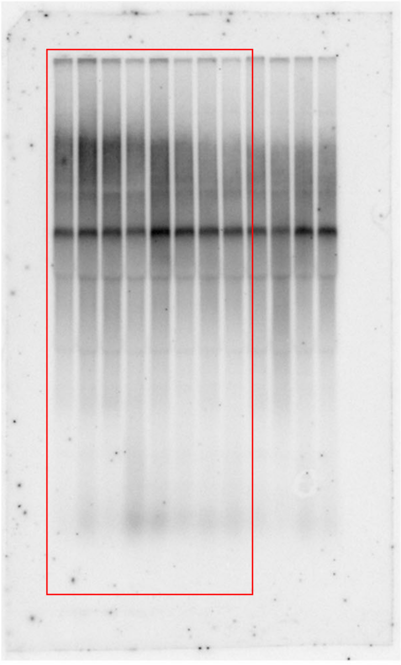

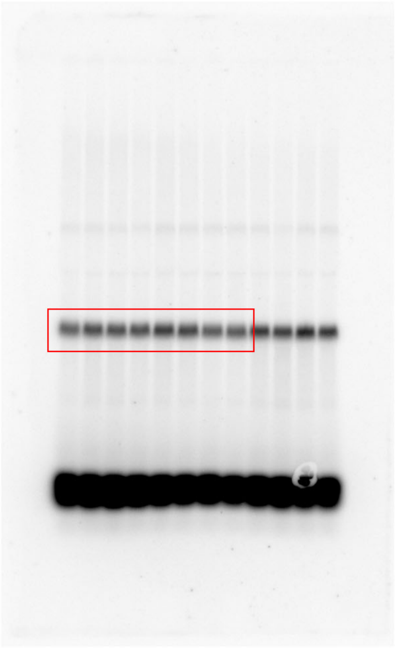

NaOH

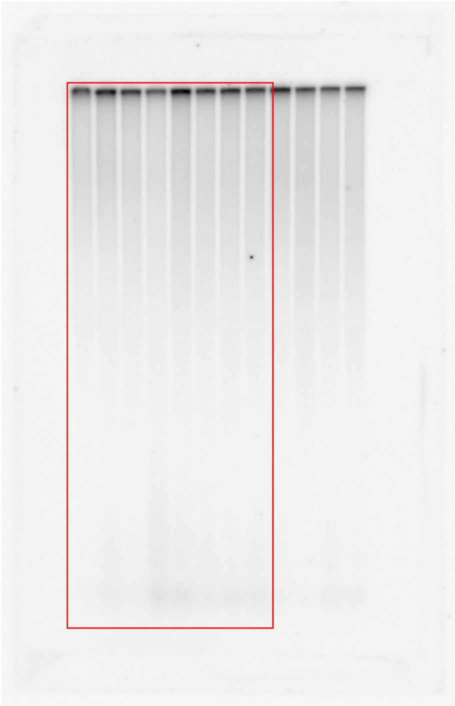

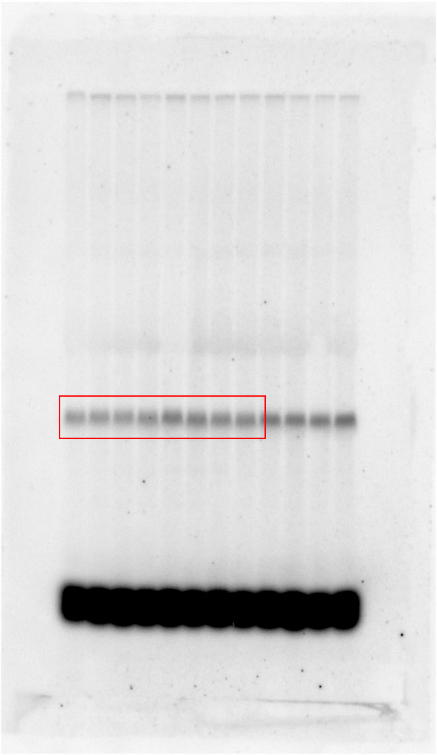

Uncropped blots (related to Supplementary Fig. 5 and 6)

Fig. S5a

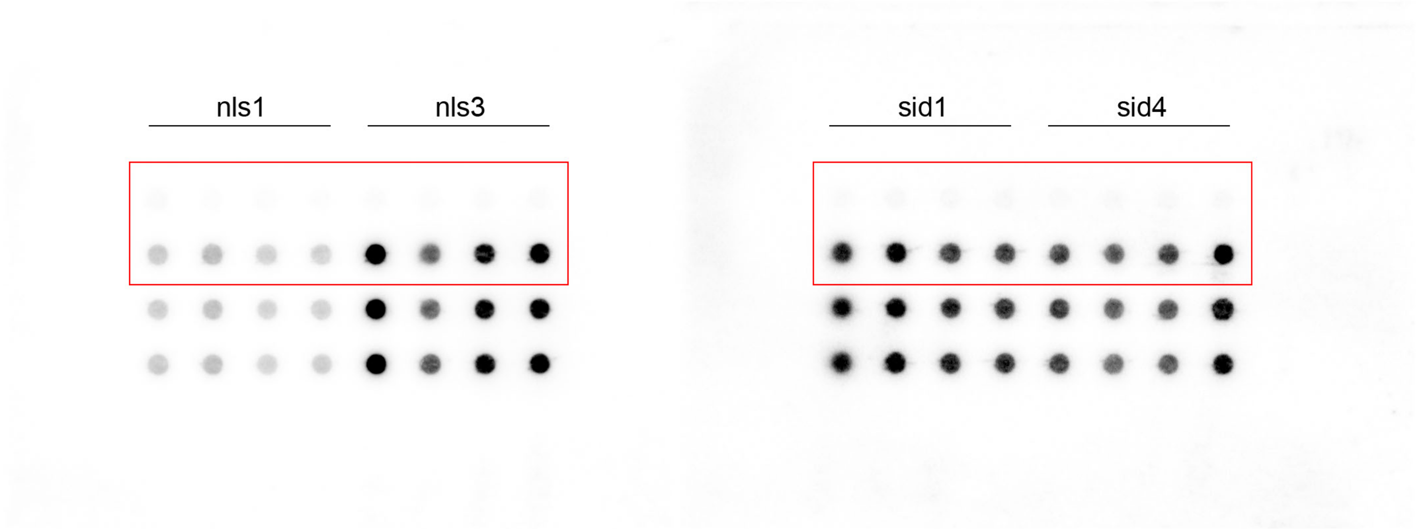

Fig. S6

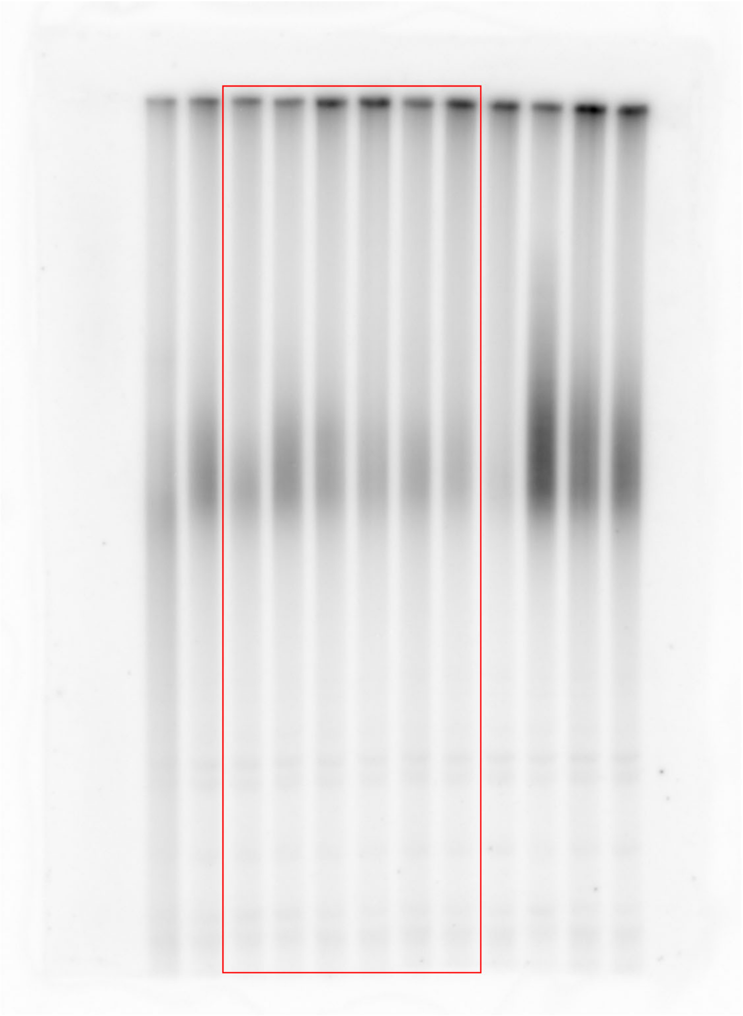

Supplement: Supplementary file 7 — Source Data [file 41467_2021_24097_MOESM7_ESM.zip › Uncropped blots.pdf]
